# Performance of Daily Pads Containing Ophytrium and Chlorhexidine Digluconate 3% in Dogs With Local Cutaneous Bacterial and/or *Malassezia* Overgrowth

**DOI:** 10.3389/fvets.2021.579074

**Published:** 2021-05-07

**Authors:** Marina Gatellet, Roxane Kesteman, Benjamin Baulez, Félix Pradiès, Pierre-Marie Cadot, Hélène Dropsy, Pierre Fiora, Jean-Loup Mathet, Elodie Ollivier, Charlotte Billy, Claudine Zemirline, Xavier De Jaeger

**Affiliations:** ^1^Ceva Santé Animale, Libourne, France; ^2^Clinique Vétérinaire VPLUS, Saint Aubin de Blaye, France; ^3^Clinique Vétérinaire SeineVet, Rouen & Boos, France; ^4^Ecole Nationale Vétérinaire d'Alfort, Maisons-Alfort, France; ^5^Clinique Vétérinaire Saint-Jacques, Agen, France; ^6^Clinique Vétérinaire des Glycines, Orléans, France

**Keywords:** chlorhexidine, Ophytrium, DOUXO S3, Pads, *Staphylococcus*, *Malassezia*, dog, overgrowth

## Abstract

Local disturbances of the microbiota are common in dogs with underlying skin conditions. Antiseptic topical products are indicated to control such superficial disorders. The objective of this study was to evaluate the performance of a daily application of pads containing Ophytrium and chlorhexidine digluconate 3% (DOUXO® S3 PYO Pads, Ceva Santé Animale, France) in dogs with focal bacterial and/or *Malassezia* overgrowth. Eighteen dogs with focal skin dysbiosis were included in the analysis of this prospective, multicentric, field study. Dogs received daily pad applications for 14 days. Bacterial and/or *Malassezia* counts per microscopic field and a global score of the most affected patch (0–17 scale based on extension, severity, bacterial, and *Malassezia* cytological scores) were assessed by a veterinarian and pruritus by the owner (Pruritus Visual Analog Scale) on days (D)0, D7, D14. Owner and veterinarian evaluations for performance and satisfaction were recorded. Eleven dogs had primarily cocci overgrowth and seven mostly *Malassezia*. Mean bacterial and *Malassezia* counts decreased after 14 days (6.9–1.1; 7.6–1.5, respectively); 88.9% of dogs achieved a ≥70% microbial decrease and had ≤2 bacteria and ≤1 *Malassezia* per oil field. Mean global score of the most affected patch and pruritus score significantly improved at D14, respectively, from 8.6 to 2.6 and 4.5 to 1.2 (*P* < 0.05 each, mean improvements of 70.4 and 71.4%, respectively). Global veterinary assessment of the protocol was satisfactory, good, or excellent in 88.9% of cases. Most owners (94.4%) considered the protocol efficacious. Using a pad containing Ophytrium and chlorhexidine digluconate 3% daily for 14 days improved the skin condition and pruritus of dogs with local dysbiosis, resulting in high satisfaction levels for both veterinarians and dog owners.

## Introduction

Many alterations in the skin surface microbiota or host defense linked to skin disorders can promote bacterial or yeast overgrowth. *Staphylococcus pseudintermedius* is the most common bacterium responsible for secondary skin conditions in dogs and cats (1), but they can also occasionally involve other *Staphylococcus* species, notably *Staphylococcus aureus* from human origin (2). Other cocci such as streptococci or rods, for example *Pseudomonas aeruginosa* or *Escherichia coli* less commonly cause skin infections (3). *Malassezia pachydermatis* is the most common yeast causing secondary skin disorders in dogs (3). There is a scientific consensus regarding the management of bacterial and/or *Malassezia* overgrowth: most recent guidance is to use topical therapy as first line and to not use systemic antimicrobials (4–6). Topical disinfectants suffices. Systemic antimicrobial agents are usually not necessary, may cause systemic adverse effects and overuse or misuse may favor and spread antibiotic resistance (7–9). Topical formulations offer a local approach, delivering ingredients directly to the skin where pathogens live. Among the wide variety of antimicrobial agents that have been used in dogs, the antiseptic chlorhexidine has the strongest evidence supporting its antibacterial action (1, 3, 10). Moderate evidence on chlorhexidine effect when used alone against *Malassezia* is available (6, 11–14) and most of them are *in vitro* data. Ophytrium is a specific purified natural extract of *Ophiopogon japonicus*, also called “Mondo grass.” It was shown *in vitro* to have an action on the mechanical, microbiological, and immunological skin barriers. It increased tight junctions, filaggrin, Natural Moisturizing Factors contents, ceramides, and limited Trans Epidermal Water Loss to strengthen the mechanical skin barrier (15). It limited the adhesion and biofilm formation of *S. aureus* and *S. pseudintermedius* (16) to restore the balance of the protective microbial flora. It decreased secretion of pro-inflammatory cytokines [TSLP, IL-8 (13), and IL-13] to reduce skin irritation.

As microbial skin colonization can sometimes develop locally, the use of antiseptic pads is a way to apply ingredients only where necessary.

The aim of this multicenter, field study was to assess the performance of the commercial Ophytrium and chlorhexidine digluconate 3% pads (DOUXO^®^ S3 PYO Pads, Ceva Santé Animale, Libourne, France) to reduce bacterial and/or *Malassezia* populations in dogs with local skin colonization.

## Materials and Methods

### Inclusion and Non-inclusion Criteria

Client-owned dogs of any sex and any breed, aged between 1 and 12 years, with 1–6 patches of local skin dysbiosis (each patch couldn't exceed 10 cm in diameter), were recruited from five veterinary clinics in France. All of the dogs were in good general condition based on a physical examination. The dysbiosis was confirmed with a cytology exam, using the tape strip technique and stained using a rapid technique (RAL 555 modified Giemsa stain, Centravet; Dinan, France). For each dog, the most affected patch at inclusion was considered for cocci and/or *Malassezia* count. Bacteria and *Malassezia* were counted on six Oil Immersion Fields (OIF) (X1000) and a mean number was calculated.

Dogs that were pregnant, lactating, or presenting with more than six patches of skin dysbiosis, with deep pyoderma, with pyoderma requiring systemic antibiotic treatment, with an autoimmune disease, with known hypersensitivity to chlorhexidine, with external parasitic infestation, with any major disease, or with clinical signs that may interact with our study were not included in this study. Exclusion criteria also included dogs that had received at least one of the following treatments:

- Treatment with short acting (<5 days) corticoids within 2 weeks prior to inclusion
- Initiation or modification of antihistamine treatment within 1 week prior to inclusion
- Treatment with antimicrobial or antifungal therapies within 4 weeks prior to inclusion
- Treatment with long acting (>5 days) corticoids within 4 weeks prior to inclusion
- Application of topical antiseptic(s) (e.g., chlorhexidine, diluted bleach bath) within 2 weeks prior to inclusion
- Application of any of the following topical products: lotions, sprays, shampoos within 7 days prior to inclusion.

### Pads Application

Commercially available pads containing Ophytrium and chlorhexidine digluconate 3% (DOUXO^®^ S3 PYO Pads, Ceva Santé Animale, Libourne, France) were applied once daily for 14 days. Each irritated patch was wiped for 10 s, which ensured the whole patch was covered with the solution. One 5-cm pad per patch was used. The pet owner applied the pads to the patches in the same order each day, starting with the most affected patch, which was determined by the veterinarian at the inclusion visit.

### Study Schedule and Evaluated Parameters

Each dog was seen three times by the veterinarian: inclusion visit (day 0), first follow-up visit (day 7), and second follow-up visit (day 14). The follow-up visits took place before the daily application of the pad(s) to avoid bias induced by recent application of the product. During each visit, a skin sample was taken, using the tape strip method and stained using a rapid technique (RAL 555 modified Giemsa stain, Centravet; Dinan, France). Microscopic examination was performed under OIF. Several parameters were recorded:

- On all the patches:

- Irritation severity score: 0–4 (0 = none; 1 = mild; 2 = moderate; 3 = severe; 4 = very severe; from Bensignor et al., 2016).
- Irritation extent score: 0–5 (0 = not present, 1 = size less than a square of 2 cm, 2 = size less than a square of 4 cm, 3 = size less than a square of 6 cm, 4 = size less than a square of 8 cm, 5 = size less than a square of 10 cm).

A transparent sheet, which had the different sized squares printed on it, was provided to the vets to help them determine the relevant score.

- On the most irritated patch (as determined by the veterinarian at the inclusion visit):

- Two semi-quantitative scores based on the cytological exam [adapted from (17)]:

- Bacteria: 0–4 (0 = no bacteria/inflammatory cell, 1 = occasional bacteria/inflammatory cells, 2 = bacteria/inflammatory cells presented in low numbers but detectable rapidly, 3 = bacteria/inflammatory cells present in large numbers and detectable without difficulties, 4 = massive amount of bacteria/inflammatory cells present and detectable rapidly without difficulties)
- *Malassezia*: 0–4 (0= no *Malassezia* yeast, 1 = occasional *Malassezia* yeasts, 2 = *Malassezia* yeasts presented in low numbers but detectable rapidly, 3 = *Malassezia* yeasts presents in large number and detectable without difficulties, 4 = massive amount of *Malassezia* yeasts present and detectable rapidly without difficulties).

- A global score to provide an overview of the evolution of this patch: the score was calculated by adding the irritation extent score, the irritation severity score, and the two semi-quantitative scores (bacteria and *Malassezia*), resulting in a score between 0 and 17.
- Cocci and/or *Malassezia* counts per OIF (x1000): mean of six OIF.

At the end of the study, each case was classified as success or failure based on its bacterial and/or *Malassezia* count at D14: protocol success was defined as the number of bacteria ≤2 per OIF and *Malassezia* count ≤1 per OIF.

- Pruritus score, using a visual analog scale (PVAS). This consisted of a 10-cm line with a scale of severity/frequency words at 2 cm intervals, on which the pet owner put a mark to indicate the level of itching of his pet. The pruritus score was collected by measuring the distance between the beginning of the line (corresponding to a normal dog) and the owner's mark.
- At the end of the study, both pet owner and veterinarian satisfaction were collected through questionnaires. The veterinarian was asked to appraise the clinical evolution of the patches of skin dysbiosis compared to D0 (Absent or worst, weak, satisfactory, good, excellent), provide a general assessment about the product application on a scale from 0 (very poor) to 5 (excellent), and indicate whether the dog needed another treatment for the initial patch(es) at the end of the protocol. The owner was asked to assess the performance, and the practicality of the protocol, as well as some characteristics of the product including the fragrance and skin hydration properties, using a four-point scale: totally disagree, somewhat disagree, somewhat agree, totally agree. They were also asked to evaluate the overall response to the product (No response, a poor response, a fair response, a good response, an excellent response) [from (18)].

### Data Analysis

Data were analyzed using SAS v9.4 (SAS Institute). The level of significance was set at *P* < 0.05. Efficacy data were summarized per visit (if applicable). For quantitative parameters, number of animals, mean, standard deviation, and for some, median, first and third quartiles were provided. Frequency distributions and number of animals were detailed for categorical variables.

Success of a case was assessed on the mean number of cocci and/or *Malassezia* per OIF evolution. It was analyzed using a wilcoxon paired test on the absolute variation at Day 14 compared to Day 0 (Count at D14–Count at D0).

The same analysis was performed for the mean number of bacteria per OIF and the mean number of *Malassezia* per OIF evolutions separately, respectively, based on animals with bacteria at Day 0 and *Malassezia* at Day 0.

Global score of the most irritated patch and pruritus evaluation (using the VAS) were analyzed using a linear mixed model with repeated measures. Visit day (Day 7, Day 14) and baseline value were fixed effects. Random effect was the animal. Ninety-five percent confidence interval and *p*-value were provided to analyse the significance of the least square means estimates.

## Results

### Demographics and Initial Characteristics of the Patches

Twenty dogs were enrolled in the study. Among them, two dogs were excluded from the final analysis: one dog was involved in a road traffic accident and required intravenous administration of amoxicillin-clavulanic acid for 2 days, and another one presented an adverse event involving also the pet owner (both owner and dog had a severe allergic reaction, so the product administration had to be discontinued). 66.7% of included dogs were female (12/18), with a mean body weight of 19.6 kg. The majority of them had a short-haired coat (11/18, 61.1%) and not dense (10/18, 55.6%).

At the start of the study, half of the included dogs presented with only one irritation patch (Table 1).

**Table 1:** Number of irritated patches at the inclusion visit (*n* = 18).

**Number of patches**	**Day 0**
0	0.0% (0)
1	50.0% (9)
2	22.2% (4)
3	16.7% (3)
4	5.6% (1)
5	5.6% (1)

Most affected patches were located on the dog's face or on the dorsum, with severity score being between 2/4 and 3/4 and extent ranging from 2.3/5 to 3.5/5 in most of the cases (Table 2).

**Table 2:** Location, extent, and severity of the most irritated patch at D0 (*n* = 18).

**Location of the most irritated patch**	**% cases (*n* = 18)**	**Initial irritation severity score Mean (*SD*) (/4)**	**Initial irritation extent score Mean (*SD*) (/5)**
Axilla	5.6% (1)	3.0 (–)	5.0 (–)
Dorsum	11.1% (2)	2.5 (0.7)	3.5 (2.1)
Face	44.8% (8)	2.8 (0.7)	2.3 (1.0)
Flank	5.6% (1)	3.0 (–)	3.0 (–)
Front leg/paw	5.6% (1)	2.0 (–)	2.0 (–)
Hind leg/paw	5.6% (1)	4.0 (–)	4.0 (–)
Tail	5.6% (1)	4.0 (–)	4.0 (–)

At day 0, 5 dogs (27.8%) had both *Malassezia* and bacterial colonization, 9 (50%) only presented with bacterial overgrowth, and 4 dogs (22.2%) only had a *Malassezia* overgrowth. Neutrophils were seen on 9/18 cases (with intracellular cocci in seven cases), which indicates a more severe dysbiosis. Macrophages were observed in three cases. Mean cocci count at day 0 was 6.9 per OIF and mean *Malassezia* count at day 0 was 7.6 yeasts per OIF. Mean global score of the most irritated patch at D0 was 8.6/17 and mean initial pruritus score was 4.5/10 (moderate itching/regular episodes) (Table 3).

**Table 3:** Main characteristics of the microbial overgrowth at D0.

**Parameter**	**Mean (*SD*)**
Global score of the most irritated patch (/17) (*n* = 18)	8.6 (2.5)
Number of cocci per OIF in affected dogs (*n* = 14)	6.9 (5.3)
Number of *Malassezia* per OIF in affected dogs (*n* = 9)	7.6 (8.5)
Pruritus score (PVAS /10) (*n* = 18)	4.5 (2.9)

### Evolution of Bacterial and/or *Malassezia* Counts

At the end of the study, 16/18 dogs (88.9%) were considered as protocol success as they presented a number of cocci ≤ 2 per OIF and *Malassezia* count ≤1 per OIF. All these dogs had a ≥70% decrease of their microbial count after 14 days of daily application.

**Figure 1:**
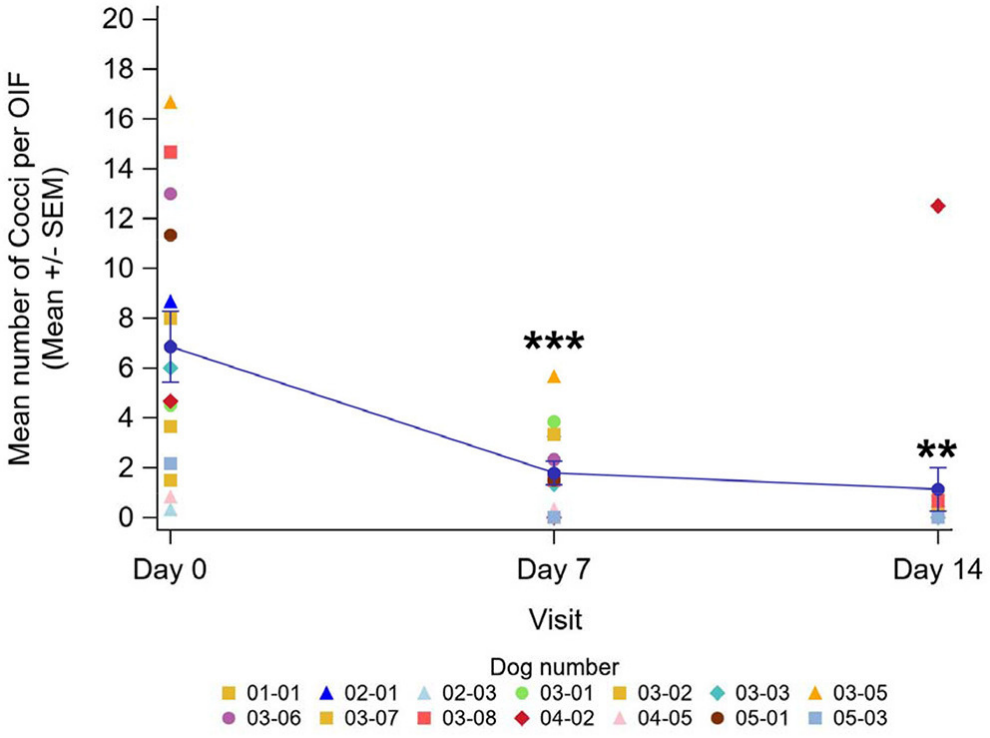
Evolution of the mean number of cocci per OIF at each visit with individual data by dog (*n* = 14)—Line plot. **Results are significantly different from D0 (*P* < 0.01). ***Results are significantly different from D0 (*P* < 0.001).

Mean number of cocci per OIF significantly decreased at all timepoints (*P* < 0.01), on average by 73.2% at D7 (from 6.9 to 1.8) and by 78.1% at D14 (mean number of cocci: 1.1 at D14) (Figure 1).

**Figure 2:**
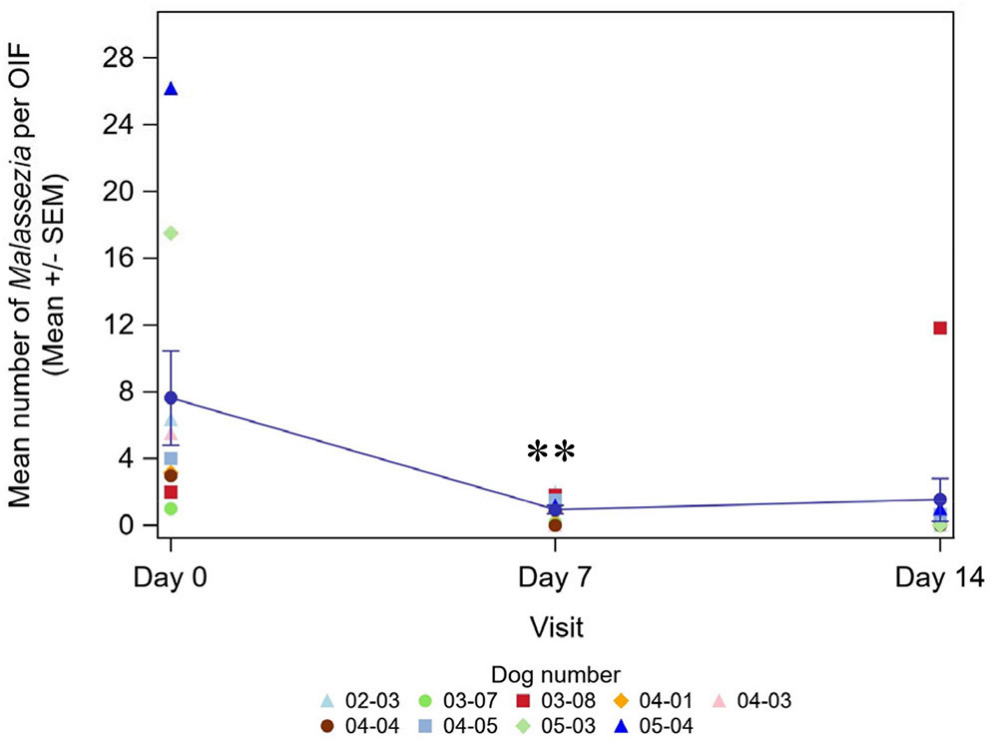
Evolution of the mean number of *Malassezia* per OIF at each visit with individual data by dog (*n* = 9)—Line plot. **Results are significantly different from D0 (*P* < 0.01).

The mean number of *Malassezia* per OIF significantly decreased after 7 days of application by 76.2% (*P* < 0.05, from 7.6 at D0 to 0.9 at D7). On Day 14, the difference was not significant anymore (mean number of *Malassezia*: 1.5 at D14, mean improvement 31.3%), due to a unique dog which had an increased *Malassezia* count (Figure 2).

There were no longer any intracellular bacteria or macrophages observed.

### Global Score of the Most Irritated Patch

The global score (an aggregate of the extent, severity, bacteria, and *Malassezia* scores) significantly decreased on average by 47.1% after 7 days of application and by 70.4% at the end of the 14-day course (*P* < 0.0001 both).

### Owner Pruritus Score (PVAS)

**Figure 3:**
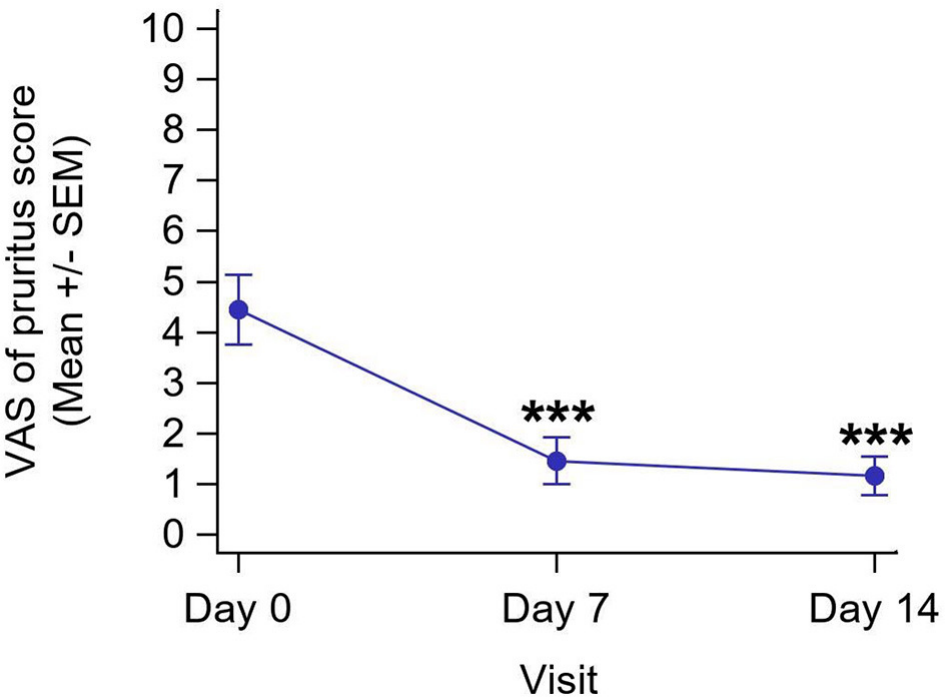
Evolution of pruritus score at each visit (*n* = 18). ***Results are significantly different from D0 (*P* < 0.001).

Mean pruritus score at inclusion was 4.5/10, representing moderate itching/regular episodes. After 7 days of application, pruritus significantly improved by 71.4% on average (*P* < 0.001) from 4.5/10 to 1.5/10, remaining stable up to the end of the protocol (mean score: 1.2/10 at D14) with a 71.4% mean improvement (Figure 3).

### Veterinarian and Owner Questionnaires at the End of the Study

Veterinarians' global judgement of the improvement compared to D0 is presented in Table 4. In 14/18 (77.8%) cases, improvement was considered as good or excellent, and in two additional cases (11.2%) as satisfactory. This result is in accordance with the 4.3/5 mean general assessment mark given about the product application, corresponding to a good to excellent opinion.

**Table 4:** Veterinarian global judgement of the improvement compared to!!break D0 (*n* = 18).

**Global judgement of the improvement compared to D0 [%(*****n*****)]**	
Absent or worst	0% (0)
Weak	11.1% (2)
Satisfactory	11.2% (2)
Good	22.2% (4)
Excellent	55.6% (10)

In 13/18 (72.2%) of cases, the 2-week topical course was considered to have been all that was required to manage the initial patch(es). In the remaining five cases, the veterinarians suggested additional measures they considered necessary to resolve the case (Table 5). For one dog, surgery of the lip was considered necessary due to saliva accumulation increasing humidity in the patch.

**Table 5:** Additional measures suggested at the end of the protocol to ensure resolution of the affected patch(es) (*n* = 5).

**1**	**Complementary antibiotics should have given quicker results**
**2**	**Bacterial overgrowth retroceded but *Malassezia* overgrowth worsened in spite of clinical improvement**
**3**	**Surgery to tighten the lip**
**4 and 5**	**Continue the same wipes application for a longer course**

**Figure 4:**
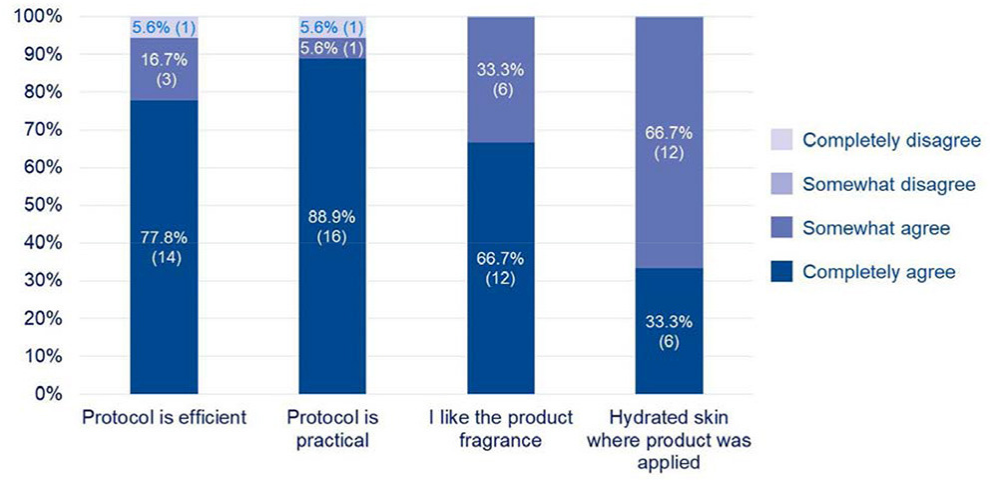
Pet owners' evaluation at the end of the protocol (*n* = 18).

Overall, pet owners considered the response to the product as good or excellent in 14/18 (77.8%) of the cases, which is in accordance with veterinarians' opinion. Two out of eighteen considered a fair response and the two remaining indicated a poor response to the products. Pet owners' evaluation on the other parameters is presented in Figure 4. They appreciated performance and practicality of the protocol (17/18, 94.4% owners each) as well as the fragrance of the product (18/18, 100%). All of them considered the skin was hydrated where the product was applied.

### Adverse Events

Three adverse events involving two cases were reported during the study:

- One dog had a road traffic accident and received systemic antibiotics (Amoxicillin-clavulanic acid via intravenous route).
- One dog had severe xerosis on patches that the product was applied to 3 days after starting the protocol.
- The owner of the above mentioned dog also developed xerosis, erythema, blisters, and then ulcerative plaques localized on the fingers used for product application.

The two dogs were withdrawn from the study.

## Discussion

The results of this prospective study provide evidence of the effectiveness of pads containing chlorhexidine digluconate 3% combined with Ophytrium to manage skin dysbiosis caused by either cocci and/or *Malassezia*. The main criterion used to consider the case as a success was the reduction of microbial count to reach ≤2 cocci per OIF and ≤1 *Malassezia* per OIF. In healthy dogs, the number of bacteria per oil field is <2 (19), justifying our criterion choice. Regarding *Malassezia*, there is no definite number to assess a *Malassezia* overgrowth, because it varies notably between the body location and breeds (19). Some veterinarians consider overgrowth to be when seeing more than four yeasts per OIF while others are more restrictive using the rule of one *Malassezia* per OIF. In this study, dogs at inclusion were considered to have *Malassezia* overgrowth when there were at least three *Malassezia* per OIF (mean calculated on six OIF). The cytological success threshold was set at one *Malassezia* per OIF as it is always possible to see a very low number of yeasts in a normal dog. This threshold is consistent with a previous report (12). In our study, 50% of dogs (9/18) fulfilled this criterion after 7 days of daily application and 88.9% of dogs (16/18) cytologically recovered according to our criterion after 14 days. These data show a quick activity of the product containing chlorhexidine digluconate 3% against both cocci and *Malassezia*, which is in accordance with other studies indicating an action of chlorhexidine against *Malassezia* (10–13). The duration of treatment required to achieve a cytological resolution was 2 weeks in the majority of the cases. According to the most recent consensus guidelines for treatment of *Malassezia* dermatitis in dogs (6), strong evidence is available only for the use of a 2% miconazole and 2% chlorhexidine shampoo, and moderate evidence is available for a 3% chlorhexidine shampoo. This study brings additional evidence that 3% chlorhexidine without azole-derivative ingredient has an action in this type of presentation.

Pruritus is a common clinical sign observed in 30–40% of dermatology consultations (20, 21) and is often a reason why pet owners seek veterinary advice. In this study, a rapid improvement of itching was observed, with owners reporting a mean decrease of 71.4% of the pruritus score after 7 days of application. Confidence interval at 95% was at this time point [−85.7; −57.0]. This reduction can be linked to the antiseptic effect of chlorhexidine digluconate at 3% but also to soothing properties of Ophytrium, which has been shown to decrease the production of pro-inflammatory cytokines *in vitro* (15).

There is no control group in this study, which is the main limitation of this trial. Nonetheless, some studies on similar clinical conditions with a placebo or a vehicle group, indicated few resolutions in the placebo group compared with the treated group after 2 weeks of application (22, 23), even with shampoos where a mechanical effect on microbial count is suspected. Another study, involving an antifungal shampoo vs. a physiologic one showed a significant difference just after one shampoo lasting up to 4 days following the application of the antifungal shampoo whereas no significant difference was observed with the physiologic shampoo (24). A study published in 2000 in dogs with *Malassezia* dermatitis using azole-derivative and vehicle shampoos showed a decrease of the yeast count with all treatments but a significantly higher number of yeasts in the vehicle group after the first week of application (25). In another study, the authors found no significant differences between an antiseptic shampoo and a physiologic one (without the ingredient with antimicrobial properties) in bacterial counts on healthy dogs or atopic dogs with no overinfection (26). This observation may be explained by the low number of bacteria found on each site and the absence of pathogenic strain (<3 bacteria per OIF irrespective of the case). In the absence of skin colonization by pathogenic bacteria, the number of bacteria per OIF is low and stable. When a pathogenic strain is present, the bacterial growth rate is much higher and not limited by the action of commensal flora. Even if a mechanical effect is present, remaining pathogenic bacteria may grow again after application of a product. It can be hypothesized that an antiseptic formulation may have a more powerful effect than a physiological one, due to the additional mechanical and antimicrobial properties.

Therefore, as natural reduction of germ levels is expected to be low, it was decided to not include a placebo group and only compare the dogs before and after the 14 day course.

We reported three adverse events in this study. Two of them concerned the same case and were probably linked to the product with an intense xerosis in a dog and xerosis with erythema going to ulcerations on fingers of its pet owner. Another person in the household who also applied the product did not show any clinical signs. Analysis of the pads (quality, microbiology did not reveal any abnormalities of the product. Skin condition of the dog improved after being treated with prednisolone 0.5 mg/kg/day for 2 days and amoxicillin-clavulanic acid 12.5 mg/kg twice a day for 10 days. Owner's skin recovered after allantoin-based cream application. The main hypothesis is an intolerance to an ingredient, and this is more likely to chlorhexidine. Intolerance or allergy to this ingredient has previously been reported in both humans and dogs (12, 27, 28). These events are uncommon if we consider the widespread use of chlorhexidine in healthcare settings.

Evolution of extent, severity of the patches, and cytological scores (bacteria and *Malassezia*) were grouped together in a global score to give an overview of the cases. This was done previously in a similar way without the extent and *Malassezia* score because not studied (22, 27). Nonetheless, if clinical and cytological evolutions go the same way, usually clinical resolution and cytological resolutions are not fully concomitant. Some publications report clinical cure precedes the cytological one (29) while some other authors report the opposite (12). Some clinical signs can take longer to resolve as they are signs of chronicity (for example hyperpigmentation, lichenification) or because of the physiological repair (12), even if cytological resolution is achieved. This paper also reported that erythema seems to decrease less than other parameters, even if the difference is not significant. This could explain why in our study after 14 days of application, some dogs were still “Mild” regarding the status of the microbial overgrowth with a satisfactory microbial count. It is currently recommended to continue antimicrobial topical application until 7 days beyond clinical resolution of all lesions (4, 19). According to our observations and previous reports, it could be recommended not to rely only on complete resolution of clinical signs but also on microbial count to decide whether or not the application of products should be stopped, especially in the case of chronic manifestations.

Repeated application of disinfectant products may dry out skin (30–32). In this study, 100% of owners stated that they thought the skin of their pet was hydrated where the product was applied, indicating that the formula allowed the skin to be hydrated. This hydration may be partly due to the inclusion in the formula of Ophytrium which has been shown to limit water loss in a model of reconstructed epidermis stressed by pro-inflammatory cytokines (15).

Chlorhexidine topical products are sometimes reported by some pet owners to have an unpleasant smell (internal survey). Even in the absence of studies assessing adherence to a protocol of patients using products with an offensive odor, it is likely that it has an effect on compliance. Covering the smell of chlorhexidine may be beneficial due to the positive effect on compliance. One veterinarian reported this point spontaneously. All pet owners involved in this study appreciated the product's fragrance, and adhered to the protocol of application.

Many formulations containing antimicrobial ingredients are available on the market but few are convenient for areas difficult to reach such as interdigital spaces or skin folds, particularly if needed to be rinsed off. Pads or wipes impregnated with antiseptic ingredients offer an interesting two-fold action: a mechanical one to remove microbes, crusts, dead cells, and a microbiological one by bringing the antiseptic ingredient (chlorhexidine in our case) directly to the skin. The additional benefit of the pads tested in the study is to help strengthen the mechanical, immunological, and microbiological skin barriers thanks to the three-fold action of Ophytrium contained in the formula (15, 16).

## Conclusion

Daily application of pads containing chlorhexidine digluconate at 3% and Ophytrium (DOUXO® S3 PYO Pads) quickly and significantly reduced cocci and *Malassezia* populations in dogs with local dysbiosis without dehydrating their skin. It also allowed a significant reduction of clinical manifestations, including pruritus, leading to high satisfaction of veterinarians and pet owners. The use of azole-derivative products in addition to chlorhexidine seems not to be necessary in this type of *Malassezia* colonization.

The scent of the product successfully covered the unpleasant chlorhexidine odor, which is probably an added value for adequate use and adherence to the protocol.

DOUXO® S3 PYO Pads are convenient and potent formulation to deliver ingredients only where necessary, or in areas difficult to reach with other formulations such as skin folds or interdigital spaces.

## Data Availability Statement

The raw data supporting the conclusions of this article will be made available on request to the corresponding author.

## Ethics Statement

The animal study was reviewed and approved by Avogadro Ethics Committee. Written informed consent was obtained from the owners for the participation of their animals in this study.

## Author Contributions

MG, RK, CZ, and XD contributed to the conception and design of the study. BB, FP, P-MC, HD, PF, and J-LM recruited dogs for the study and performed clinical and cytological examinations. CB participated in results elaboration. MG, XD, and CB wrote the first draft of the manuscript. All authors contributed to the article and approved the submitted version.

## Conflict of Interest

MG, RK, EO, CB, CZ, and XD are employees of Ceva Santé Animale. The remaining authors received fees from Ceva Santé Animale.

## Abbreviations

D: day
OIF: oil immersion field
PVAS: pruritus visual analog scale.
